# Efficacy of Ozonated Water, Chlorine, Chlorine Dioxide, Quaternary Ammonium Compounds and Peroxyacetic Acid Against *Listeria monocytogenes* Biofilm on Polystyrene Surfaces

**DOI:** 10.3389/fmicb.2018.02296

**Published:** 2018-10-12

**Authors:** Ahmed Mahmoud Korany, Zi Hua, Tonia Green, Ines Hanrahan, Saadia Helmy El-Shinawy, Adel El-kholy, Gamal Hassan, Mei-Jun Zhu

**Affiliations:** ^1^School of Food Science, Washington State University, Pullman, WA, United States; ^2^Food Hygiene and Control Department, Faculty of Veterinary Medicine, Beni-Suef University, Beni Suef, Egypt; ^3^Washington Tree Fruit Research Commission, Wenatchee, WA, United States

**Keywords:** biofilm, *L. monocytogenes*, sanitizers, peroxyacetic acid, polystyrene, organic matter

## Abstract

*Listeria monocytogenes* contaminated processing equipment and the general packing environment have been implicated in deadly foodborne listeriosis outbreaks, highlighting the significance of proper sanitization and disinfection of food contact surfaces. This study aims to comprehensively evaluate antimicrobial efficacy of commercially available, economical sanitizers at practical concentrations against *L. monocytogenes* biofilm formed on polystyrene surfaces under different conditions. Ozonated water 1-min treatment at 1.0, 2.0, and 4.0 ppm resulted in ∼0.9, 3.4, and 4.1 log reduction of *L. monocytogenes* single strain biofilm grown on polystyrene surfaces, respectively. However, its efficacy was dramatically diminished in multi-strain *L. monocytogenes* biofilm and was further compromised by aged biofilm and in the presence of organic matter. Quaternary ammonium compounds (QAC) at 100/400 ppm, chlorine at 100/200 ppm, chlorine dioxide at 2.5/5.0 ppm and peroxyacetic acid (PAA) at 80/160 ppm resulted in 2.4/3.6, 2.0/3.1, 2.4/3.8, and 3.6/4.8 log reduction of *L. monocytogenes* single strain biofilm, respectively. Antimicrobial efficacies of all tested sanitizers against 7-day-old biofilm were much lower when compared to 2-day-old biofilm, with PAA being the least influenced by the age of the biofilm. Organic matter conditioning with diluted milk or apple juice dramatically impacted the antimicrobial efficacy of all sanitizers. PAA treatment of 1 min at 160–200 ppm resulted in a 3.2–3.5 log reduction against 7-day-old biofilm in the presence of organic matter, thus showing its effectiveness in eradicating *L. monocytogenes* biofilm on polystyrene surface. Collectively, data highlight the importance of timely and thoroughly cleaning food contact surfaces before disinfection and provides practical information and guidance for the food industry in selecting the most effective sanitizer in their sanitizing regimes to eliminate *L. monocytogenes* biofilm.

## Introduction

*Listeria monocytogenes* causes nearly 1,600 illnesses in the United States annually with a 16% or higher mortality rate (Scallan et al., 2011). It is frequently incriminated in ready-to-eat meat (CDC, 1989, 1999, 2000; Currie et al., 2015), soft cheese (Heiman et al., 2016), and fresh produce (McCollum et al., 2013; Angelo et al., 2017) associated listeriosis outbreaks. It has a strong ability to form biofilms on a wide variety of abiotic surfaces commonly used in food processing facilities (Di Bonaventura et al., 2008), persisting on food processing equipment and food contact surfaces and representing a continuous contamination source (Almeida et al., 2013). Cross-contamination of foods with pathogens via contaminated equipment and contact surfaces is an important factor contributing to foodborne outbreaks (Gaulin et al., 2012; Heiman et al., 2016). The fresh produce processing equipment and environment have been implicated as important contamination sources for *L. monocytogenes* outbreaks (McCollum et al., 2013; Angelo et al., 2017). Thus, it is critical to effectively clean and sanitize all equipment and food-contact surfaces to eliminate *L. monocytogenes* in these niches.

Biofilm is composed of multiple cells attaching to the surface, which collectively produce protective extracellular polymeric substances (Buchanan et al., 2017). *L. monocytogenes* in biofilms displayed greater resistance to antimicrobial sanitizers than planktonic cells (Frank and Koffi, 1990; Norwood and Gilmour, 2000; Robbins et al., 2005). The susceptibility of *L. monocytogenes* in biofilm to disinfectants is influenced by a number of factors including strain characteristics and composition (Stopforth et al., 2002; Borucki et al., 2003; Kostaki et al., 2012; Poimenidou et al., 2016), the growth stage or age of biofilm (Stopforth et al., 2002; Saa Ibusquiza et al., 2011), the type of surfaces (Krysinski et al., 1992) as well as the cleanliness and/or the food residues/organic matter level of those surfaces (Gram et al., 2007; Saa Ibusquiza et al., 2011). Food residues/organic matter can affect bacterial biofilm formation as well as bacterial resistance to the applied sanitizers (Gram et al., 2007).

Food contact surfaces are cleaned and disinfected routinely in apple packing and similar ready-to-eat produce handling facilities. Chlorine, quaternary ammonium compounds (QAC), and peroxyacetic acid (PAA) are EPA registered and economical sanitizers commonly used for surface antimicrobial intervention in the food industry including the apple industry. Due to safety concerns about the production of carcinogenic halogenated by-products resulting from chlorinated organic compounds (Parish et al., 2003), chlorine dioxide has been used as an alternative to chlorine (Mahmoud et al., 2008). It has a higher oxidation capacity than chlorine (∼2.5 times) (Benarde et al., 1965). Ozone is a potent antimicrobial effective at relatively low concentrations with no residual effects, and with a broad-spectrum antimicrobial activity (Moore et al., 2000; Khadre et al., 2001), making it an ideal choice as a final sanitizer for food contacting surfaces.

Although chemical sanitizer intervention against *L. monocytogenes* biofilm on surfaces has been extensively studied, studies on their practical efficacy against biofilm under different conditions are limited. Most of sanitizers were used at a concentration much higher than practical or recommended levels (Gram et al., 2007; Minei et al., 2008; Belessi et al., 2011; Poimenidou et al., 2016). In addition, different *L. monocytogenes* strains, conditions, sanitizers, surfaces as well as different enumeration methodologies were used in different papers (Gram et al., 2007; Minei et al., 2008; Belessi et al., 2011; Poimenidou et al., 2016), making it impossible to provide guidance on effective sanitizers for industrial application. In the present study, using a calibrated biofilm enumeration method, we attempted to comprehensively evaluate the antimicrobial efficacy of commonly used chemical sanitizers in the food industry at practical application times and concentrations. Single- and 6-strain *L. monocytogenes* biofilms at different stages (age) of formation on polystyrene surfaces were tested and compared in the presence or absence of organic matter. Diluted apple juice and milk were used as organic matter to simulate persistent surface contamination on surface niches that are difficult to access and clean in a wide range of processing environments.

## Materials and Methods

### *Listeria monocytogenes* Strains and Cultures Preparation

*Listeria monocytogenes* strain NRRL B-33069 (1/2a, bovine milk isolate), NRRL B-33466 (1/2b, environmental isolate), NRRL B-57618 (1/2a, human clinical isolate), NRRL B-33006 (1/2b complex, garlic isolate), NRRL B-33053 (4b, coleslaw isolate), and NRRL B-33385 (4b, human clinical isolate) were used to prepare a single strain and 6-strain cocktail inoculum. These strains were provided by the culture collection of the National Center for Agricultural Utilization Research (NRRL), USDA Agricultural Research Service (Peoria, IL, United States) and were stored at -80°C in Trypticase Soy Broth [Becton, Dickinson and Company (BD), Sparks, MD, United States] with 0.6% (w/v) yeast extract (Fisher Scientific, Pittsburgh, PA, United States) (TSBYE) and glycerol (20%, v/v). Each culture was twice-activated in TSBYE consecutively at 35 ± 2°C for 24 h statically, then pelletized by centrifuging and suspended in Modified Welshimer’s broth (MWB, HiMedia, West Chester, PA, United States) or TSB to achieve the target population density at ∼10^7^ CFU/ml. To prepare a 6-strain cocktail, each individual strain was activated separately, then combined at equal volumes.

### Biofilm Formation

The above prepared culture of a single strain or 6-strain cocktail was transferred into each well of a sterile 96-well microtiter plate (polystyrene, Costar, Corning, NY, United States) conditioned with or without organic matter. Wells loaded with MWB or TSB broth were used as a negative control. The plates were covered with lid and incubated at 22°C (room temperature, RT) for 2, 4, and 7 days to grow *L. monocytogenes* biofilms with different ages. For each independent study, there were 6 wells per treatment. Triple independent experiments were conducted for each treatment combination.

### Organic Matter Conditioning

Organic matter/soil level of the food contact surface dramatically impacted *L. monocytogenes* adherence ability and its resistance to sanitizers (Papaioannou et al., 2018). To simulate harborage sites within processing plants that cannot be easily cleaned, the wells were pretreated with 100 μl of 1:10 diluted apple juice or whole milk for 5 min at RT, then drained and dried in the biosafety cabinet for 30 min prior to being subjected to *Listeria* biofilm growth and sanitizer treatments.

### Biofilm Assessment by Crystal Violet Staining

Following 2 or 4 days of incubation at RT, single strain *L. monocytogenes* (NRRL B-33069) biofilm grown in a 96-well polystyrene plate was washed with sterile water three times to remove loosely attached bacteria. The plate was air-dried at 37°C for 30 min, followed by an addition of 150 μl of 1% (w/v) crystal violet solution to each well and incubation at 22°C for 45 min. The wells were triple washed with sterile water, air-dried for 30 min at 37°C, and the crystal violet stain was dissolved by incubating with 100 μl of ethyl alcohol (95%) for 30 min at RT. The solubilized crystal violet was measured at 595 nm (CV-OD_595_) using a Synergy H4 microplate Reader (BioTek^®^ Instrument, Inc.). Each treatment had six replicates in a particular experiment, and the studies were repeated three times independently.

### Live/Dead Staining of Biofilm

The biofilms formed on wells of 96-well polystyrene plate were subjected to the BacLight Live/Dead^®^ staining kit (Molecular Probes) per the manufacture’s manual. Briefly, cells in biofilms were incubated with 1:1 mixture of SYTO 9 and propidium iodide dyes in the dark for 15 min, rinsed with phosphate buffered saline, pH 7.4 (PBS), and then visualized using EVOS FL fluorescence microscope (Life Technologies). The excitation/emission for SYTO 9, the green-fluorescent stain, is 480/500 nm and denotes live cells with intact membranes. The excitation/emission for propidium iodide, the red-fluorescent stain, is 490/635 nm and denotes dead cells with damaged membranes.

### Biofilm Dislodgement and Enumeration

Biofilm formed in a 96-well polystyrene plate was subjected to triple washing with sterile PBS. After adding 100 μl of sterile PBS, biofilm in respective wells was dislodged either mechanically by scraping vigorously using a sterile tip or ultrasonically (Pitts et al., 2003). To detach biofilm ultrasonically, the 96-well plate was subjected to sonication treatment (40 KHz SharperTek^®^ ultrasonic, Pontiac, MI, United States) for 1 min at 22°C, unless specified (Chavant et al., 2004), followed by pipetting and mixing thoroughly. The dislodged cells (100 μl) from each well were transferred to a 1.5 ml micro-centrifuge tube for 10-fold serial dilutions. The appropriate dilutions were plated on TSAYE plates in duplicate and incubated at 35 ± 2°C for 48 h.

### Biofilm Intervention Using Chemical Sanitizers

After washing three times with 150 μl of sterile PBS, biofilm cells were subjected to 100 μl of the respective sanitizer treatment at selected concentration at 22°C for 1 min. After discarding the sanitizer, 150 μl of Dey-Engley Neutralizing Broth (D/E) (Oxoid, United States) was added to neutralize any sanitizer residues. After washing two times with PBS, biofilm with or without sanitizer treatment was detached with 100 μl of sterile PBS ultrasonically and enumerated as described above. For each independent study, there were six wells per treatment. Triple independent experiments were conducted for a selected treatment.

Bioside HS (EnviroTech, Modesto, CA, United States) containing 15% PAA was used to prepare 80, 160, and 200 ppm PAA solutions. The concentration of PAA was confirmed using a titration kit (AquaPhoenix Scientific, Hanover, PA, United States). Chlorine solution (100 and 200 ppm) was prepared from Accu-Tab (Pace international, Wapato, WA, United States), and the pH was adjusted to 6.8 with 6 M HCl. Quaternary ammonium compound (QAC) solutions (100–400 ppm) were prepared from STOPIT (Pace International, United States). Chlorine dioxide solutions were generated on-site using a customized chlorine dioxide generator (Pace International). The concentrations of chlorine dioxide solutions were calibrated using the chlorine dioxide Palintest kit (Palintest^®^, Golden, CO, United States). Ozonated water was generated on-site using a Guardian Ozone generator (Guardian Manufacturing Inc., Cocoa, FL, United States). The concentration of ozonated water solution was calibrated using a titration kit (High Level Ozone Test Kit, Guardian Manufacturing Inc., Cocoa, FL, United States) and used at 1.0, 2.0, and 4.0 ppm.

### Statistical Analysis

Data were presented as mean values ± standard error of the mean averaged from three independent experiments. Analysis of variance (ANOVA) was applied to determine significant differences between different groups at *P* ≤ 0.05, using a SPSS package for Windows (Version 16).

## Results

### *L. monocytogenes* Biofilm Formation and Quantification

**FIGURE 1:**
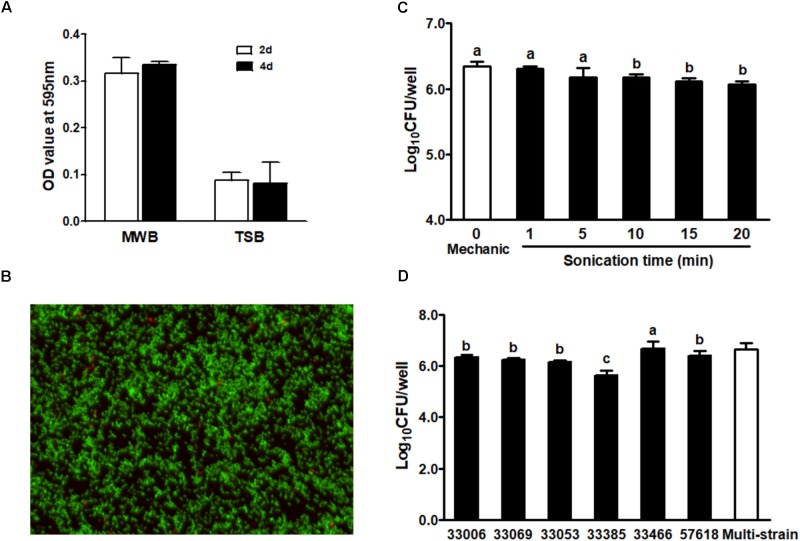
*Listeria monocytogenes* single strain biofilm on polystyrene surface incubated at room temperature. **(A)** Quantification of single strain *L. monocytogenes* (NRRL B-33069) biofilm grown in MWB or TSB for 2 or 4 days by crystal violet staining; **(B)** BacLight Live/Dead staining of 4-day-old biofilm (NRRL B-33069) grown in MWB. Images were observed under fluorescence microscope at 100× magnification. Green, live cells; red, dead cells; **(C)** Enumeration of 2-day-old biofilm (NRRL B-33069) dislodged by mechanic scraping and sonication methods; **(D)** Enumeration of 2-day-old biofilm of different *L. monocytogenes* strains by sonication. 33006 (NRRL B-33006, 1/2b complex, garlic isolate), 33069 (NRRL B-33069, 1/2a, bovine milk isolate), 33053 (NRRL B-33053, 4b, coleslaw isolate), 33385 (NRRL B-33385, 4b, human clinical isolate), 33466 (NRRL B-33466, 1/2b, environmental isolate), and 57618 (NRRL B-57618, 1/2a, human clinical isolate). Mean ± SEM, ^a-c^Bars topped with the same letter are not different at *P* ≤ 0.05. Experiments were repeated independently three times.

The single strain *L. monocytogenes* biofilms were grown on polystyrene surfaces in different media for 2 or 4 days. The density of *L. monocytogenes* biofilm was markedly higher in MWB compared to TSB, while the population size of 4-day-old biofilm was similar to that of 2-day-old biofilm regardless of growth media (**Figure 1A**). A representative live and dead staining image of 4-day-old single strain biofilm is shown in **Figure 1B**, where green fluorescence indicates live cells and red fluorescence shows dead cells in the biofilm.

The method for biofilm dislodgement affects data interpretation of the antimicrobial intervention efficacy. Therefore, we compared mechanical-scraping vs. 1-min sonication to dislodge cells of a 2-day-old biofilm and found that both methods had a similar detachment efficiency (**Figure 1C**). Sonication at 1–5 min resulted in similar CFU of *L. monocytogenes* biofilm, while sonication beyond 5 min decreased CFU enumeration of removed cells (**Figure 1C**), indicating potential cell damage during prolonged sonication. Therefore, the 1-min sonication removal method was used in the subsequent studies. We further compared the biofilm formation ability among six *L. monocytogenes* strains. There was no clear link between biofilm formation and the serotype of the selected strains (**Figure 1D**). NRRL B-33385, a 4b human clinic isolate had the lowest population density in the biofilm, while the *L. monocytogenes* environmental isolate (NRRL B-33466) showed the highest biofilm forming ability among all strains tested (**Figure 1D**).

### Single Strain *L. monocytogenes* Biofilm Inactivation by Different Sanitizers

**FIGURE 2:**
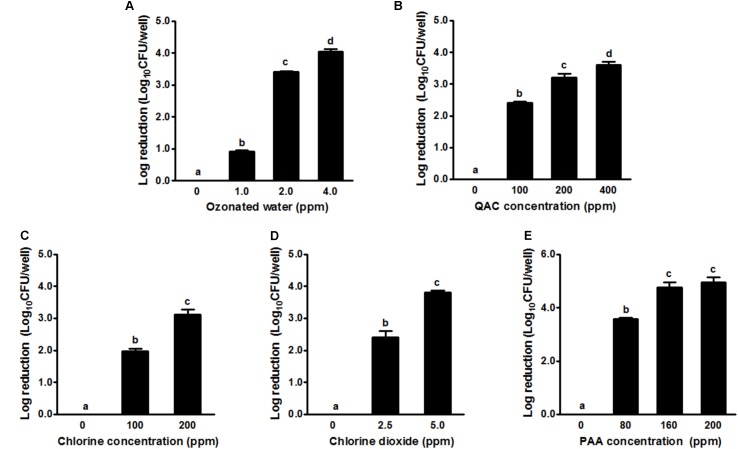
Antimicrobial efficacy of selected sanitizer interventions against 2-day-old single strain *L. monocytogenes* biofilm. **(A)** Ozonated water; **(B)** Quaternary ammonium compounds (QAC); **(C)** Chlorine; **(D)** Chlorine dioxide; **(E)** Peroxyacetic acid (PAA). Mean ± SEM, ^a-d^Bars topped with the same letter are not different at *P* ≤ 0.05. Experiments were repeated independently three times.

In general, the 2-day-old single strain *L. monocytogenes* biofilm was susceptible to all tested sanitizers (**Figure 2**). Treatment with ozonated water for 1 min at 1.0, 2.0, and 4.0 ppm resulted in a ∼0.9, 3.4, and 4.1 log reduction of single strain biofilm grown on polystyrene surfaces, respectively (**Figure 2A**). QAC 1-min intervention at 100, 200, and 400 ppm resulted in a ∼2.4, 3.2, and 3.6 log reduction of biofilm, respectively (**Figure 2B**). Chlorine 1-min intervention at 100/200 ppm and chlorine dioxide at 2.5/5.0 ppm resulted in ∼2.0/3.1 and ∼2.4/3.8 log reduction of single strain *L. monocytogenes* biofilm, respectively (**Figures 2C,D**). PAA 1-min treatment at 80 and 160 ppm achieved ∼3.6 and ∼4.8 log reduction of *L. monocytogenes* in single strain biofilm, respectively (**Figure 2E**). For each tested sanitizer, increasing concentration significantly enhanced their efficacy (**Table 1**).

**Table 1:** Log reduction of 6-strain biofilm on polystyrene surface conditioned with or without organic matter post sanitizer treatments.

Sanitizers	Conc.	2d	2d-apple	2d-milk	7d	7d-apple	7d-milk
Ozonated water	2.0 ppm	1.58 ± 0.14^Aa^	0.12 ± 0.03^Ba^	0.20 ± 0.06^Ba^	0.26 ± 0.05^Ba^	0.11 ± 0.04^Ba^	0.16 ± 0.02^Ba^
	4.0 ppm	2.27 ± 0.09^Abc^	0.36 ± 0.05^Ba^	0.31 ± 0.05^Ba^	0.70 ± 0.04^Cb^	0.24 ± 0.05^Bab^	0.29 ± 0.03^Ba^
Chlorine	100 ppm	1.91 ± 0.12^Aab^	1.24 ± 0.05^BCbc^	1.33 ± 0.09^Bb^	0.98 ± 0.11^BCDbc^	0.86 ± 0.10^CDc^	0.71 ± 0.03^Db^
	200 ppm	2.81 ± 0.14^Acd^	2.51 ± 0.10^Adf^	2.57 ± 0.11^Ac^	1.72 ± 0.11^Bd^	1.61 ± 0.12^Bd^	1.42 ± 0.13^Bcd^
Chlorine dioxide	2.5 ppm	2.31 ± 0.06^Abc^	0.90 ± 0.09^Bb^	0.63 ± 0.04^CDad^	0.87 ± 0.02^BCb^	0.52 ± 0.01^Db^	0.50 ± 0.05^Dab^
	5.0 ppm	3.55 ± 0.10^Ae^	2.23 ± 0.06^Bdef^	1.48 ± 0.07^CDb^	1.73 ± 0.07^Cd^	1.31 ± 0.07^Dd^	1.24 ± 0.08^Dc^
QAC	200 ppm	2.41 ± 0.25^Abc^	1.87 ± 0.08^ABce^	1.16 ± 0.24^BCbd^	1.35 ± 0.13^BCcd^	0.86 ± 0.05^Cc^	0.75 ± 0.08^Cb^
	400 ppm	3.06 ± 0.07^Ade^	2.20 ± 0.08^Bdef^	2.09 ± 0.05^Bc^	2.18 ± 0.10^Be^	1.56 ± 0.05^Cd^	1.44 ± 0.07^Ccd^
PAA	80 ppm	3.48 ± 0.08^Ae^	2.69 ± 0.19^Bf^	2.15 ± 0.11^Cc^	3.29 ± 0.05^Af^	2.13 ± 0.05^Ce^	1.66 ± 0.10^Dd^
	160 ppm	4.76 ± 0.06^Af^	3.62 ± 0.15^BDg^	3.70 ± 0.09^Be^	4.34 ± 0.02^Cg^	3.36 ± 0.08^BDf^	3.17 ± 0.08^CDe^
	200 ppm	4.99 ± 0.09^Af^	3.85 ± 0.16^BDg^	3.91 ± 0.18^Be^	4.46 ± 0.06^Cg^	3.50 ± 0.05^BDf^	3.31 ± 0.09^CDe^

*2d, 2-day-old biofilm; 7d, 7-day-old biofilm. PAA, peroxyacetic acid; QAC, Quaternary ammonium compounds. Mean ± SEM. Experiments were repeated independently three times. Apple:10 diluted apple juice was used as organic matter; milk:10 diluted milk was used as organic matter. Means ± SEM. ^*A-D*^Means within a row with no common letter differ significantly (*P* ≤ 0.05). ^*a-f*^Means within a column with no common letter differ significantly (*P* ≤ 0.05)*.

### Factors Influencing Antimicrobial Efficacy of Selected Sanitizers Against Multi-Strain *L. monocytogenes* Biofilm

**FIGURE 3:**
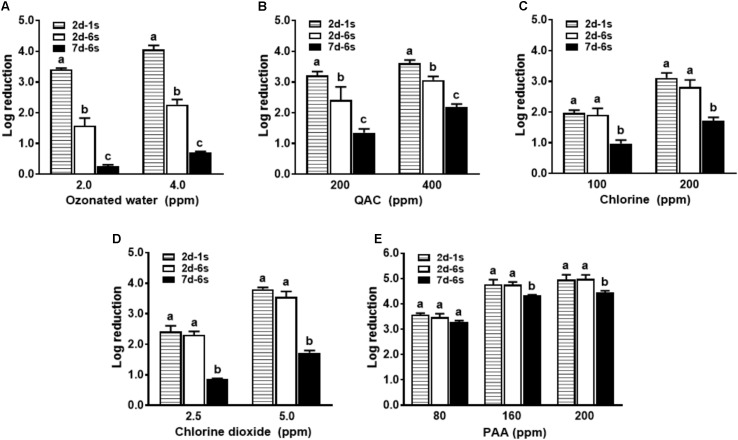
Antimicrobial efficacy of selected sanitizer interventions against *L. monocytogenes* biofilms at different ages. 2d-1s, 2-day-old single strain biofilm; 2d-6s, 2-day-old 6-strain biofilm; 7d-6s, 7-day-old 6-strain biofilm. **(A)** Ozonated water; **(B)** Quaternary ammonium compounds (QAC); **(C)** Chlorine; **(D)** Chlorine dioxide; **(E)** Peroxyacetic acid (PAA). Mean ± SEM, ^a-c^Bars topped with the same letter at the same concentration are not different at *P* ≤ 0.05. Experiments were repeated independently three times.

Antimicrobial efficacy of ozonated water and QAC against a mixed strain *L. monocytogenes* biofilm was reduced by 1.8 log and 0.6–0.8 log, respectively, when compared to single strain *L. monocytogenes* biofilm of the same age (**Figure 3**). However, chlorine, chlorine dioxide and PAA showed similar bactericidal effects against the 6-strain biofilm compared to single strain biofilm of the same age (**Figure 3**).

Antimicrobial efficacies of all sanitizers against 7-day-old biofilm was diminished when compared to 2-day-old biofilm (**Figure 3** and **Table 1**), indicating mature biofilm had a higher resistance to sanitizer interventions. Antimicrobial efficacy of ozonated water and chlorine dioxide was the most influenced by the age of the biofilm (**Figures 3A,D**). There was about a 1.6 and 1.8 log net reduction for 4.0 ppm ozonated water and 5.0 ppm chlorine dioxide, respectively, and a 1.0 log net reduction of biocidal effects for chlorine and QAC when treating the 7-day-old biofilm (**Figures 3A–D**). The efficacy of PAA was relatively less impacted by the age of the biofilm (**Figure 3E**). There was only 0.2 and 0.5 log net reduction for 80 ppm and 160–200 ppm PAA, respectively (**Figure 3E** and **Table 1**).

**FIGURE 4:**
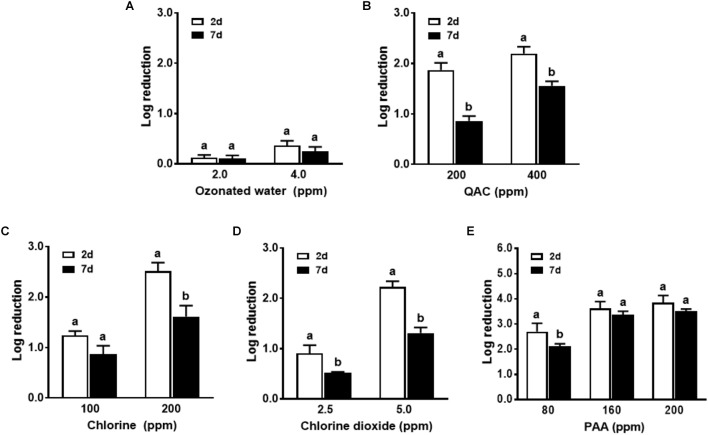
Antimicrobial efficacy of selected sanitizer interventions against 6-strain *L. monocytogenes* biofilm on polystyrene surfaces in the presence of organic matter from apple. 2d, 2-day-old biofilm; 7d, 7-day-old biofilm. **(A)** Ozonated water; **(B)** Quaternary ammonium compounds (QAC); **(C)** Chlorine; **(D)** Chlorine dioxide; **(E)** Peroxyacetic acid (PAA). Mean ± SEM, ^a,b^Bars topped with the same letter at the same concentration are not different at *P* ≤ 0.05. Experiments were repeated independently three times. 1:10 diluted apple juice was used as organic matter.

**FIGURE 5:**
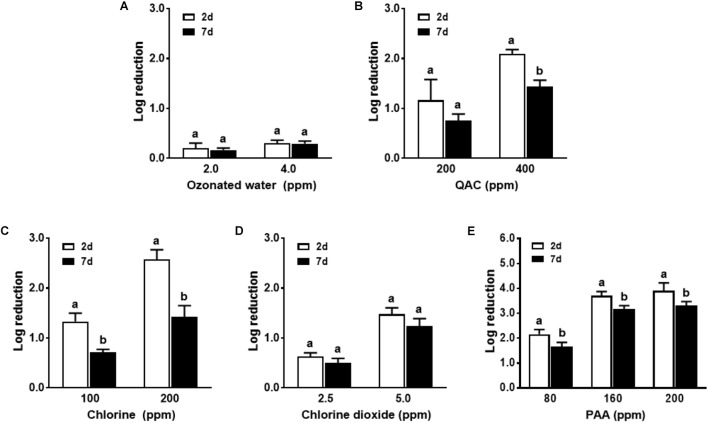
Antimicrobial efficacy of selected sanitizer interventions against 6-strain *L. monocytogenes* biofilm on polystyrene surfaces in the presence of organic matter from milk. 2d, 2-day-old biofilm; 7d, 7-day-old biofilm. **(A)** Ozonated water; **(B)** Quaternary ammonium compounds (QAC); **(C)** Chlorine; **(D)** Chlorine dioxide; **(E)** Peroxyacetic acid (PAA). Mean ± SEM, ^a,b^Bars topped with the same letter at the same concentration are not different at *P* ≤ 0.05. Experiments were repeated independently three times. 1:10 diluted whole milk was used as organic matter.

Conditioning of the surface with organic matter of apple origin further counter-acted the biocidal effects of all sanitizers against biofilm regardless of biofilm age (**Figure 4** and **Table 1**). Ozonated water had a minimal efficacy against *L. monocytogenes* in both 2-day-old and 7-day-old biofilm in the presence of organic matter with apple origin (**Figure 4A**). Chlorine at 200 ppm, 5.0 ppm chlorine dioxide, and 400 ppm QAC were able to reduce 6-strain *L. monocytogenes* biofilm by 1.6, 1.3, 1.6 log, respectively, for 7-day-old biofilm, and by 2.5, 2.2, and 2.2 log, respectively, for 2-day-old biofilm (**Figures 4B–D**). PAA 1-min treatment at 160–200 ppm resulted in 3.4–3.5 log reduction against 7-day-old biofilm conditioned with organic matter from apple origin (**Figure 4E** and **Table 1**). Similarly, surface conditioning with organic matter from milk origin impaired antimicrobial intervention against both 2-day-old and 7-day-old biofilm. Soiling from milk caused slightly more net reduction in biocidal efficacy in general compared to organic matter from apple origin (**Figure 5** and **Table 1**).

## Discussion

### Strain Variability in Biofilm Formation and Quantification

*Listeria monocytogenes*, as a true environmental species, is prevalent in many produce-associated locales and operations. Food processing equipment and environment have been identified as important contamination sources for cantaloupe and caramel apple listeriosis outbreaks (McCollum et al., 2013; Angelo et al., 2017). This highlights the importance of proper and effective sanitization of food contact surfaces.

Different *L. monocytogenes* strains have distinctive abilities to form biofilm (Borucki et al., 2003). To compensate the strain variability of biofilm formation and antimicrobial resistance to individual sanitizer a 6-strain *L. monocytogenes* biofilm containing three serotypes that have caused the majority of human listeriosis (1/2a, 1/2b, and 4b) (Silk et al., 2012) from different food and environmental sources were used in this study. As reported in a previous study (Borucki et al., 2003), there was a strain variability in biofilm formation. However, there was no clear relationship between biofilm and serotype, which is consistent with a previous report (Borucki et al., 2003).

### Antimicrobial Efficacy of Ozonated Water Against *L. monocytogenes* Biofilm Formed Under Different Conditions

Since its approval by FDA to be directly applied to food, in storage and during processing (Rice and Graham, 2001), ozone in both the aqueous and gaseous phase has been increasingly used in the food industry (Moore et al., 2000; Baumann et al., 2009; Sheng et al., 2018). Ozone, known for its high oxidation capacity and potent antimicrobial activity, reacts with organic material 3000 times faster than chlorine (EPRI, 1997), and has the ability to attack microbial cell membranes, causing cell lysis and death. Ozonated water showed a strong efficacy against a 2-day-old single strain *L. monocytogenes* biofilm. A 1-min of 4.0 ppm treatment resulted in a 4.1 log reduction of biofilm, which was similar or higher than 200 ppm chlorine, 400 ppm QAC, and 5.0 ppm chlorine dioxide at the same exposure time. Similarly, 1-min 1.0 ppm ozonated water exposure resulted in 4.2 log reduction in a 4-day-old single strain *L. monocytogenes* biofilm on stainless steel (Baumann et al., 2009). However, the bactericidal effect of ozonated water against multi-strain *L. monocytogenes* biofilm was dramatically reduced and further compromised by increased age and soiling by organic matter from apple or milk origin. In support of our findings, the presence of organic matter from either milk or meat broth decreased the net log reduction of gaseous ozone (4.0 ppm for 4 h) against *Listeria innocua*, a non-pathogenic surrogate of *L. monocytogenes*, biofilm on the stainless steel surface from ∼3.0 log reduction to less than 1.0 or even 0.5 log reduction, respectively (Moore et al., 2000). The reduced disinfection rate of ozonated water against the 6-strain and/or the aged biofilm was likely due to the complexity of biofilm structure or exopolysaccharide layer of the aged and/or multi-strain biofilm, and thus poor penetration or reaction with ozone. It might also be due to differential antimicrobial sensitivities of different stages of *L. monocytogenes* in biofilm (Chavant et al., 2004). The counter-acting/suppressive effects of organic matter on ozone efficiency is likely due to their high reactivity with ozone thus competing with bacteria. It might also result from physical shielding effects of food residues. However, the organic matter soiling had a less impact on the efficacy of ozone against *Staphylococcus aureus* biofilm on stainless steel surface (Moore et al., 2000). Collectively, although ozone is a potent antimicrobial, its bactericidal activity is compromised against the aged 6-strain biofilm in the presence of organic matter, highlighting practical challenges and the importance of intelligent application of ozonated water for surface disinfection. It is highly desirable for ozonated water disinfection to be preceded by more frequent and effective cleaning to remove food residue and organic matter to maximize its efficacy.

### Antimicrobial Efficacy of Chlorine, Chlorine Dioxide, QAC, and PAA Against *L. monocytogenes* Biofilm Formed Under Different Conditions

Similar to the ozonated water, QAC was less effective against the 6-strain biofilm compared to single strain biofilm, but multi-strain had a minor impact on antimicrobial efficacy of chlorine, chlorine dioxide and PAA. The antimicrobial efficacy of sanitizers against mature biofilm was decreased by 0.6–1.8 log depending on sanitizers and their concentrations. Besides ozonated water, chlorine dioxide efficacy was most significantly impacted by the age of biofilm. In support, 200 ppm QAC/chlorine, or 150 ppm PAA at a 2-min contact were found to be less bactericidal against the bacteria in 7-day-old or 14-day-old biofilm of total microbial or mixed-strain (including *L. monocytogenes* and background microflora), on stainless steel surface conditioned with beef wash rinse than 2-day-old biofilm (Stopforth et al., 2002). Increased resistance against nisin, a bacteriocin, and PAA was seen in 11-day-old biofilm compared to a 4-day-old *L. monocytogenes* biofilm (Saa Ibusquiza et al., 2011). Similarly, resistance of *L. monocytogenes* in biofilm on stainless steel surface against 4640 ppm chlorine, 20000 ppm PAA, and 10000 ppm QAC was considerably higher with increased age of biofilm (Belessi et al., 2011).

Soiling of polystyrene surfaces with diluted milk or apple juice further reduced bactericidal effects of all sanitizers. In combination, PAA is the most effective sanitizer treatment against the aged biofilm on the soiled polystyrene surface, which achieved a 3.2–3.5 log reduction after 1-min exposure to 160–200 ppm PAA. In agreement with our data, PAA at a range of 80 to 320 ppm was shown to be more effective than the same concentration of chlorine in inactivation of *L. monocytogenes* in a 2-day-old *L. monocytogenes* and *Pseudomonas* spp. multi-species biofilm in the presence of milk origin organic matter (Fatemi and Frank, 1999). Organic matter conditioning, especially from meat emulsion, greatly counteracted biocidal effects of PAA, chlorine and QAC against *L. monocytogenes* biofilm on stainless steel surface (Gram et al., 2007).

The antimicrobial efficacy of PAA and QAC detected in this study was higher or comparable to the previous studies, where 5-min exposure to 2000 ppm PAA and 500 ppm QAC led to a 1.5–3.6 and 1.4–3.7 Log CFU/cm^2^ reduction of 3-day-old *L. monocytogenes* biofilm on polystyrene surface, respectively, depending on *L. monocytogenes* strain (Poimenidou et al., 2016). A 1-min and 10-min exposure to 7.0 ppm chlorine dioxide resulted in a 2.3 and 3.7 Log CFU/cm^2^ reduction of 4-day-old 5-strain *L. monocytogenes* biofilm on stainless steel coupons, respectively (Vaid et al., 2010), in contrast to 3.6 and 1.7 Log CFU/well reduction of 2-day-old and 7-day-old biofilm on polyester surface, respectively, caused by 1-min exposure to 5.0 ppm chlorine dioxide.

Data collaboratively indicated that PAA tended to be a viable sanitizer for the polystyrene surface disinfection in the food industry. The bactericidal effects of PAA might be due to its high reactivity and oxidizing power in combination with its low molecular size and high decomposition rate, which facilitates its penetration into biofilm matrix (Saa Ibusquiza et al., 2011). Biocidal effects of PAA against biofilm of *L. monocytogenes* and other foodborne pathogens on different processing and food-contact surfaces warrant further research. It was reported that *L. monocytogenes* biofilm on stainless steel surface had a lower population density and a higher susceptibility to PAA and QAC disinfection compared to polystyrene surface (Poimenidou et al., 2016).

## Conclusion and Perspectives

In general, all sanitizers tested in this study at the concentrations commonly used in the food industry showed a strong bactericidal effect against 2-day-old single strain *L. monocytogenes* biofilm. PAA at 160–200 ppm showed a larger net log reduction compared to other sanitizers tested. The antimicrobial efficacy of all sanitizers was impaired when the biofilm was aged, and/or a soiled surface was present. This diminished effect was the most significant for ozonated water, followed by chlorine dioxide, QAC and chlorine, while PAA was the least influenced. A 1-min treatment of 160–200 ppm PAA resulted in 3.2–3.5 log reduction of the aged biofilm on polystyrene surface with organic matter.

Our data illustrate the importance of establishing and maintaining a good cleaning process prior to sanitizer disinfection in the food industry. Food residues or soiling by organic matter, regardless of sources, diminished efficiency of sanitizers against pathogenic bacteria in general, although some sanitizers are less impacted. It is highly desirable to clean and remove organic matter/soiling effectively prior to sanitizer disinfection to maximize their efficacy. Results from this study also reflected the significance of periodical application of sanitizers to avoid establishment of the aged biofilm, which was much more difficult to be eradicated compared to the fresh one. This study provides critical and practical information for the food industry in selecting sanitizers in their surface disinfectant regimes for delicate processing niches.

## Author Contributions

AK and M-JZ designed the experiments. AK, ZH, and TG performed the experiments. M-JZ and AK wrote and revised the manuscript. IH, SE-S, AE-k, and GH edited the manuscript.

## Conflict of Interest Statement

The authors declare that the research was conducted in the absence of any commercial or financial relationships that could be construed as a potential conflict of interest.
